# Patient-led development of digital endpoints and the use of computer vision analysis in assessment of motor function in rare diseases

**DOI:** 10.3389/fphar.2022.916714

**Published:** 2022-09-12

**Authors:** Elisa Ferrer-Mallol, Clare Matthews, Madeline Stoodley, Alessandra Gaeta, Elinor George, Emily Reuben, Alex Johnson, Elin Haf Davies

**Affiliations:** ^1^ Aparito Ltd, Wrexham, United Kingdom; ^2^ Duchenne UK, London, United Kingdom

**Keywords:** video, computer vision analysis, Duchenne muscular dystrophy, digital biomarkers, digital endpoints, rare diseases

## Abstract

Digital health technologies are transforming the way health outcomes are captured and measured. Digital biomarkers may provide more objective measurements than traditional approaches as they encompass continuous and longitudinal data collection and use of automated analysis for data interpretation. In addition, the use of digital health technology allows for home-based disease assessments, which in addition to reducing patient burden from on-site hospital visits, provides a more holistic picture of how the patient feels and functions in the real world. Tools that can robustly capture drug efficacy based on disease-specific outcomes that are meaningful to patients, are going to be key to the successful development of new treatments. This is particularly important for people living with rare and chronic complex conditions, where therapeutic options are limited and need to be developed using a patient-focused approach to achieve the biggest impact. Working in partnership with patient Organisation Duchenne UK, we co-developed a video-based approach, delivered through a new mobile health platform (DMD Home), to assess motor function in patients with Duchenne muscular dystrophy (DMD), a genetic, rare, muscular disease characterized by the progressive loss of muscle function and strength. Motor function tasks were selected to reflect the “transfer stage” of the disease, when patients are no longer able to walk independently but can stand and weight-bear to transfer. This stage is important for patients and families as it represents a significant milestone in the progression of DMD but it is not routinely captured and/or scored by standard DMD clinical and physiotherapy assessments. A total of 62 videos were submitted by eight out of eleven participants who onboarded the app and were analysed with pose estimation software (OpenPose) that led to the extraction of objective, quantitative measures, including time, pattern of movement trajectory, and smoothness and symmetry of movement. Computer vision analysis of video tasks to identify voluntary or compensatory movements within the transfer stage merits further investigation. Longitudinal studies to validate DMD home as a new methodology to predict progression to the non-ambulant stage will be pursued.

## 1 Introduction

Digital biomarkers are increasingly gaining attention and being used alongside traditional biomarkers in clinical trials for rare diseases (Servais et al., 2021; Davies et al., 2022) and common conditions (Coran et al., 2019; Stephenson et al., 2020; Viceconti et al., 2020; Demeyer et al., 2021; Stephenson et al., 2021). Digital biomarkers are “objective, quantifiable, physiological and behavioural measures that are collected by means of digital devices that are portable, wearable, implantable or digestible” (Babrak et al., 2019). The ability to capture patient-generated data in a continuous, longitudinal fashion, through digital technology, combined with the use of automated analysis for data interpretation can reduce bias. In addition, this, together with the potential to reduce patient burden from on-site hospital visits, provides a more holistic picture of how the patient feels and functions in the real world.

Partnership with patient communities in the co-design and co-development of digital biomarkers is key to address unmet patient needs and to fill in the regulatory gaps for the effective use of new digital endpoints in drug development. Aparito’s Patient Group Accelerator Programme aims to identify disease-specific hallmarks meaningful to patients living with rare and complex chronic conditions^1^. Through its distinctive approach in capturing and measuring these hallmarks, using the Atom5™ platform, the programme aims to impact clinical trials, by giving greater disease insights of what’s important to patients and to improve patient’s trial experience by alleviating the burden of hospital visits through remote monitoring.

In the work described herein, Aparito partnered with Duchenne UK^2^, the leading UK medical research charity focussed on Duchenne muscular dystrophy (DMD), founded by parents of children living with DMD, and with global reach into the different stakeholders involved in the development of innovative treatments for DMD. The collaboration aimed at developing a digital platform to enable innovative outcome measures for DMD. The project involved a small group of patients to explore the feasibility and usability of a tailored digital solution and to gather their thoughts on the approach, and exploratory data that could indicate its potential clinical utility as new digital endpoints for DMD.

DMD is a genetic muscle-wasting disease, caused by various mutations in the gene that codes for dystrophin, a protein that ensures muscle integrity. Lack of dystrophin causes muscles to be damaged far more easily and to lose their function progressively. DMD almost exclusively affects boys, as the dystrophin gene is located on the X-chromosome. The current prevalence is between one in 3,500 and one in 5,000 live male births. The condition leads to progressive muscle inflammation, damage and loss of strength and function of proximal muscles first and distal muscles later in the course of the disease. Eventually the damage to muscles results in paralysis, and patients often require assistance with eating and breathing in the latter stages of the disease. Patients are often able to retain their speech and use of their fingers well into adult life. Life expectancy is currently less than 40 years for most patients (Duan et al., 2021).

Currently, there is no cure for DMD, although corticosteroid treatment can be used to slow the progression of the disease. Other therapies aimed at restoring dystrophin function have been approved in recent years globally, although with limited success, as they are intended for use in ambulant DMD patients with specific mutations that only account for a small percentage of the DMD patient population (Yao et al., 2021).

Duchenne UK established Project HERCULES in response to the lack of evidence for health technology assessment (HTA) for DMD treatments that fully reflected the experience of families and carers (Crossley et al., 2019). Project HERCULES revised the natural history model of DMD and identified aspects of the disease not well reflected previously in the published literature or in the data collected in clinical practice (Broomfield et al., 2020). Being able to bear weight and support transfers is essential for patients and families, as the loss of this ability triggers the need for additional support (e.g., mobile aids, home adaptations). This transition from the ambulant to non-ambulant stage (Transfer Stage) has an impact on patients’ clinical care and caregiver burden, limiting their quality of life significantly. Existing tests to assess disease progression and drug efficacy, like the 6-min walk test (6MWT) (McDonald et al., 2013), the North Star Ambulatory Assessment (NSAA) (Mayhew et al., 2011), and the Performance of the Upper Limb (PUL) (Mayhew et al., 2013) test fail to capture the transfer stage sufficiently to reflect its importance to patients. Both the NSAA and the 6MWT assess patients who are still ambulant, while the PUL has been developed to assess patients who have reached the non-ambulatory stage of the disease. The role of upper limb strength and movement to support transfer is also not well defined. The Motor Function Measurement-32 (MFM-32) can be administered to both ambulant and non-ambulant patients and has shown sensitivity to change over time, which makes it interesting for capturing disease progression and loss of ambulation (Vuillerot et al., 2010).

Prior to Covid-19, these tests and scales were performed in the clinical setting and needed to be evaluated by qualified clinical experts. Frequent hospital visits create a significant burden on patients and carers, with a negative impact on quality of life. In addition, the current assessments do not allow for a continuous, holistic assessment of patients in the real-world and through the different stages of the disease. There is a need for validated outcome measures that can capture patient-generated data and that are sensitive to detect small changes in disease progression and that can reliably be used to assess the efficacy of new therapies. Patients support the development of remote assessments and of new endpoints that target a broader patient population and that allow evaluation of the maintenance of independence in daily life activities (CTTI, 2016). Systematic introduction of such measures in clinical drug development will provide much needed real-world evidence for regulatory, health technology assessment (HTA) and payer decision making (Crossley et al., 2019).

The number of approved DMD therapies is very low compared to the number of compounds that have been investigated over the past years. The reasons for this high failure rate are varied, but the lack of sensitive outcome measures appears to have an important role in a disease with high phenotypic variability and variable disease trajectory (Markati et al., 2021). Recently, the regulatory qualification of the Stride Velocity 95th Centile (SV95C) as the first digital clinical-outcome measure captured via a wearable device as a secondary endpoint, has paved the way for the wider acceptance of digital endpoints in drug efficacy clinical trials (Servais et al., 2021, 2022). In addition to wearables and sensors, video capture (through smartphone applications) appears as a feasible alternative to generate patient data in the home environment, allowing for continued data generation and objective analysis. Video-based assessments are used to evaluate functional status in DMD patients. In particular, the Duchenne Video Assessment (DVA) measures physical function in DMD patients through a series of standardised tasks assessing motor function (walking, standing, etc) and performance of daily life activities (dressing, eating, etc), recorded by parents/carers using a smartphone application (White et al., 2019) (Contesse et al., 2021). Video-based evidence has recently supported several orphan medicine approvals both in the United States and in Europe (Davies et al., 2022). However, in all these instances assessment of the videos was done by central assessors, hence restricting the analysis to subjective human evaluation. Automated video analysis might offer more in-depth parameters that can be evaluated at scale, especially when evaluating mobility and movement, and reduce bias derived from subjective assessments.

Computer vision is a field of artificial intelligence that offers great potential in human pose estimation and movement analysis (Ng et al., 2020). Computer vision-based software applications can map different parts of the body on video recordings, hence being able to estimate the position of those body parts without the need of special sensors. Computer vision analysis allows the extraction of objective, quantitative measures from videos, beyond what is possible by visual assessment alone (Davies et al., 2022). Pose estimation algorithms allow for real-time tracking and quantitative assessment of human movement and offer great potential for use in clinical evaluations (Stenum et al., 2021).

Building on Project HERCULES findings, the present work aimed at identifying new digital biomarkers that could help provide a more accurate mapping of the transfer stage in DMD and be used to measure the efficacy of new treatments. The objective of this project was to explore the feasibility of a fit-for-purpose patient-facing app to allow video recordings at home and the acceptance of patients and carers to carry out the defined tasks. We also wanted to define movement parameters and test pose estimation software to potentially assess motor performance. The use of home/community-based objective measurements in clinical drug development may lead to efficacy results that better reflect the added value of new treatments and provide robust real-world evidence for HTA and reimbursement decisions.

## 2 Methods

This project was co-designed by Aparito and Duchenne UK representatives, as part of the Aparito’s Patient Group Accelerator Programme which drives partnerships between Aparito and patient advocacy groups to advance the co-design and co-development of digital endpoints that are meaningful to patients.

Using a co-creation approach, Aparito, Duchenne UK and Project HERCULES representatives met to evaluate Atom5™ capabilities and to identify which endpoints would be most beneficial to the DMD community, to inform future research efforts and innovative clinical trial outcomes. A particular focus was on the identification of digital biomarkers to capture change during the transfer stage, while incorporating upper limb movement. Discussions with parents of DMD patients helped to inform the development of the app, and to define the upper and lower limb tasks to be performed by patients, the number of videos to be recorded and the angle of recording. A platform prototype was co-developed and its usability tested by participants and their families. At the end of the 7-day prototype testing period, a debrief meeting with parents of DMD patients was held, and a questionnaire to gather information about the platform’s ease of use and user experience was issued to all participants.

### 2.1 Atom5™ digital platform and video capture capabilities

Atom5™ digital platform supported a patient-facing mobile app, including an electronic patient reported outcome (ePRO), video capture capabilities, and an analytics dashboard. The mobile app DMD Home was configured according to the specifications co-developed with Duchenne UK patient representatives. It featured one ePRO, specifically the newly developed DMD-QoL quality of life questionnaire (Powell et al., 2021) and one module to capture video data. Participants received a QR code via email that, once scanned with their phone, allowed them to download the project specific app. Once onboarded, participants watched a basic training video to learn how to perform each task and then proceeded to record the task that had been assigned to them according to their abilities (see Participant engagement and cohorts). Once recorded, the videos were submitted and viewed on the analytics dashboard only by the staff performing the analysis. Participant data was stored in a secured server in the United Kingdom (Microsoft Azure).

### 2.2 Task selection

A meeting with patients, parents and carers of DMD patients was held to determine the physical capabilities of patients and the types of movement that patients were able to complete and record using the mobile app that were representative of the transition stage. The motor function tasks selected were: 1) walking, 2) hands-to-head while sitting, 3) hands-to-head while standing and, 4) sit-to-stand then hands-to-head while standing. Special focus was given to record upper body movements as this can be performed until relatively late in the disease but can also be measured across the disease trajectory from ambulant to non-ambulant. Table 1 shows a summary of all parameters measured within the different tasks.

**TABLE 1:** Parameters measured.

Walk		Hands to head(sitting)		Hands to head (standing)		Sit to stand	
Front	Side	Front	Side	Front	Side	Front	Side
Time	Time	Time (lift/lower/total)	Time (lift/lower/total)	Time (lift/lower/total)	Time (lift/lower/total	Time (stand/sit/total)	Time (lift/lower/total
Foot placement	Step length (scaled)	Arm area on lift (elbows forward or back)		Arm area on lift (elbows forward or back)		Foot placement	
	Heel and toe lift (scaled)	Right and left arm area on lift (symmetry of movement)		Right and left arm area on lift (symmetry of movement)		Distance between feet (scaled)	
		Shoulder location/movement		Shoulder location/movement		Leg alignment (angle at outer knee)	
		Smoothness of movement (from elbow location)		Smoothness of movement (from elbow location)			
				Foot placement			
				Distance between feet (scaled)			

#### 2.2.1 Walking

Participants were instructed to walk 10 steps. For the side view, participants were filmed in two different ways: 1) camera moving alongside the participant so that the angle is constant and 2) camera remaining stationary and panning to keep the participant in view.

#### 2.2.2 Hands-to-head while sitting

Participants were instructed to begin with hands resting on the knees, then to raise their hands onto their head, and then to lower their hands again. There was some inconsistency across participants in the angle that the videos were filmed. Some were filmed at the level of the seated child, others from an elevated angle.

#### 2.2.3 Hands-to-head while standing

Participants were instructed to stand with their arms lowered, then they were asked to raise their hands onto their head, and finally to lower their hands again.

#### 2.2.4 Sit-to-stand followed by hands-to-head

Participants were instructed to stand up from a chair, then place their hands on their head, lower their hands, and finally to sit again.

### 2.3 Participant engagement and cohorts

Duchenne UK enabled the engagement with all participants, i.e., boys living with DMD (who performed the tasks and answered the DMD-QoL questionnaire) and their parents (who used the app and video-recorded the tasks). Participants signed a consent form that detailed the duration of the prototype testing period and considerations about data privacy. Eleven participants onboarded to the app (installed and scanned their QR-code to activate the app). When participants first entered the app, they were asked an onboarding question: “To ensure we ask you to capture the best videos for you please tell us which of the following options best describes your current abilities?”: 1) “Able to walk around independently,” 2) “Requires support to walk but can stand independently,” and 3) “Unable to stand independently.” According to their answers, participants were assigned into cohort 1 (ambulatory stage), cohort 2 (transfer stage), and cohort 3 (non-ambulatory stage), respectively.

Participants self-reported how able they were, and the difference between cohort 1 and 2 is subjective in nature, meaning that participants of the same or very similar ability may end up in different cohorts. Depending on which cohort the participant was in, they were asked to do different tasks to avoid asking them to perform tasks they were unable to do. Cohort 1 participants were given all the tasks. Cohort 2 participants were given the “hands-to-head while standing” and “hands-to-head while sitting” tasks. Cohort 3 participants were only given the “hands-to-head while sitting” task. For each assigned task, participants were required to submit at least one video filmed facing the camera and one filmed from the side view.

One healthy individual (sibling to one participant living with DMD) also participated in recording of all four tasks.

### 2.4 Computer vision analysis

**FIGURE 1:**
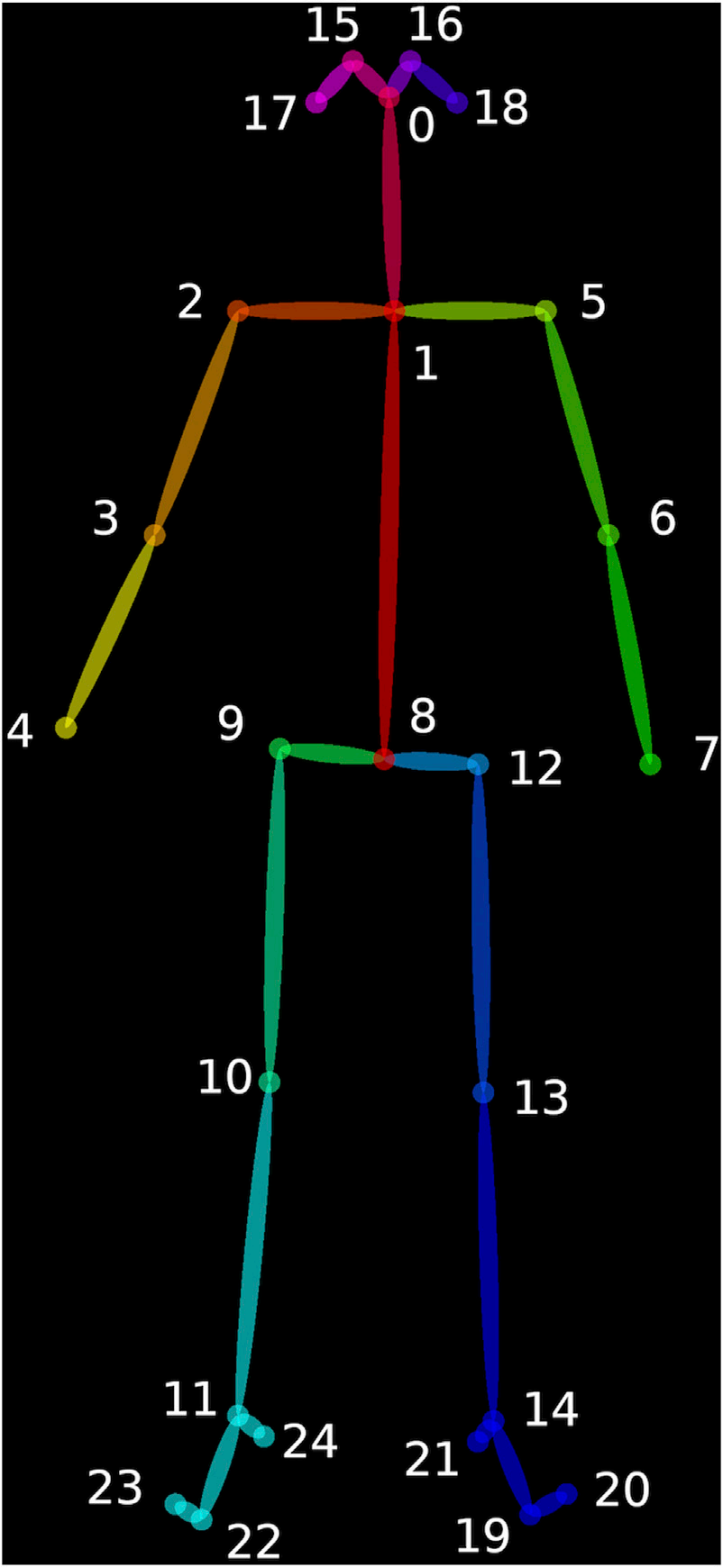
Open Pose software maps 25 points on the body including shoulders, elbows, wrists, hips (+mid-hip), knees, ankles, heels, big toes, little toes, eyes, ears, and nose (Cao et al., 2021).

Video analysis was performed using OpenPose, an open-source software that maps 25 points on the body including shoulders, elbows, wrists, hips (+mid-hip), knees, ankles, heels, big toes, little toes, eyes, ears, and nose (Figure 1) (Cao et al., 2021). OpenPose can track each of these points over the length of a video, providing time-series data that allows analysis of the capabilities or performance of participants on a task. For each task, we identified which points, or combination of points, would be most useful for analysis. OpenPose analysis of video capture data results in information of different parameters, such as trajectory, smoothness and symmetry of movement, and voluntary or compensatory movements. Data from the videos of DMD participants was compared to data from the healthy control.

### 2.5 DMD-QoL questionnaire

Project HERCULES also developed a quality-of-life measure (DMD-QoL) that maps the health stages defined in the revised natural history model (Powell et al., 2021). DMD-QoL questionnaire was configured into the DMD Home app and tested as part of this project. The adapted electronic version was approved by Oxford Innovation (OI), the DMD-QoL license holder. To test the most appropriate formatting of the questionnaire, participants were randomly assigned to one of two configurations of the app; scrolling down the screen to access additional questions or having one question per page and a “next” button to advance through them. The relative benefits of the two formats were assessed by comparing the response times for each configuration, and through a question in the study feedback questionnaire about the number of options participants thought there were under each question.

## 3 Results

### 3.1 Participants demographics and task performance

Eleven participants onboarded to the app. Median age was 13 (range 4–21, inter-quartile range: 10). Six participants were assigned to cohort 1 (ambulant) and five to cohort 3 (non-ambulant). No participants assigned themselves to cohort 2 (transfer stage). Ten participants completed the DMD-QoL questionnaire and eight uploaded at least one video of themselves doing the tasks. Some participants did not upload all the videos that they were asked to complete. A total of 62 videos were submitted by the eight participants, and 52 of these were used in the analysis (Table 2). The videos that were excluded did not include the participants completing the task correctly and/or in full view. One participant did not send data of any kind. The results below specify which view was analysed in each case. In addition, one healthy control participant performed all tasks and sent a total of seven videos.

**TABLE 2:** Summary of tasks per patient cohort and data received.

Cohort	Tasks completed	Number of videos		
		Total	Analysed	Excluded
Cohort 1 (n = 6^a^)	Walking	3 (front)	3 (front)	
		3 (front)	3 (side)	
	Hands-to-head while standing	8 (front)	7 (front)	1 (front)
		8 (side)	7 (side)	1 (side)
	Hands-to-head while sitting	9 (front)	8 (front)	1 (front)
		8 (side)	8 (side)	
	Sit-to-stand then hands-to-head while standing	5 (front)	5 (front)	
		4 (side)	4 (side)	
Cohort 3 (*n* = 5^b^)	Hands-to-head while sitting	8 (front)	3 (front)	5 (front)
		6 (side)	2 (front)	4 (side)
Control (*n* = 1)	Walking	2 (front)	1 (front)	
	Hands-to-head while standing	1 (front)	1 (front)	
		1 (side)	1 (side)	
	Hands-to-head while sitting	1 (front)	1 (front)	
		1 (side)	1 (side)	
	Sit-to-stand then hands-to-head while standing	1 (front)	1 (front)	
		1 (side)	1 (side)	
	Number of questionnaires			
Cohorts 1 and 3	DMD QoL questionnaire	10		

a Cohort 1: Participants 1, 2, 4, 7, 8 and 9.

b Cohort 3: Participants 3, 5, 6, 10 and 13.

### 3.2 Tasks and parameters measured

The task “hands-to-head while standing” was only performed by ambulant participants in cohort 1 and therefore not shown in the results, as the same parameters were measured in “hands-to-head while sitting,” which was performed by participants in both cohorts. For this exploratory stage of the analysis, we did not compare performances of the same participant on the seated and standing version of the hands-to-head task. Similarly, in “sit-to-stand followed by hands-to-head,” we did not analyse the hands-to-head part of this task as it provided no additional insight to the independent hands-to-head task.

### 3.3 Walking

We received a total of three front view and three side view videos, from two different participants, with an additional front view video from the control participant.

#### 3.3.1 Time

Participant 1 (age 7) completed the walking task twice for both the front and side view, with average times of 7.1 and 7.3 s, respectively. Participant 2 (age 13) completed the task in 6.2 and 5.8 s, from the front and side view, respectively. The control participant (age 9) completed the task in 5.5 s.

#### 3.3.2 Step length

**FIGURE 2:**
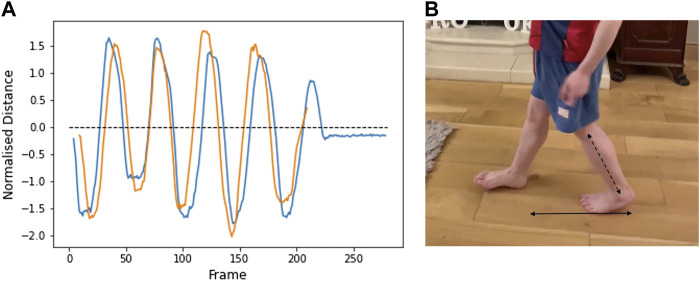
**(A)** Horizontal heel-to-heel distance over the duration of two separate videos from Participant 1 (age 7). Positive values: right heel is in front of left heel. Negative values: Right heel is behind left heel. Peaks represent the extent of each step. **(B)** Bottom arrow shows the plotted heel-to-heel horizontal distance. Distances are normalised to lower leg length (diagonal arrow).

**FIGURE 3:**
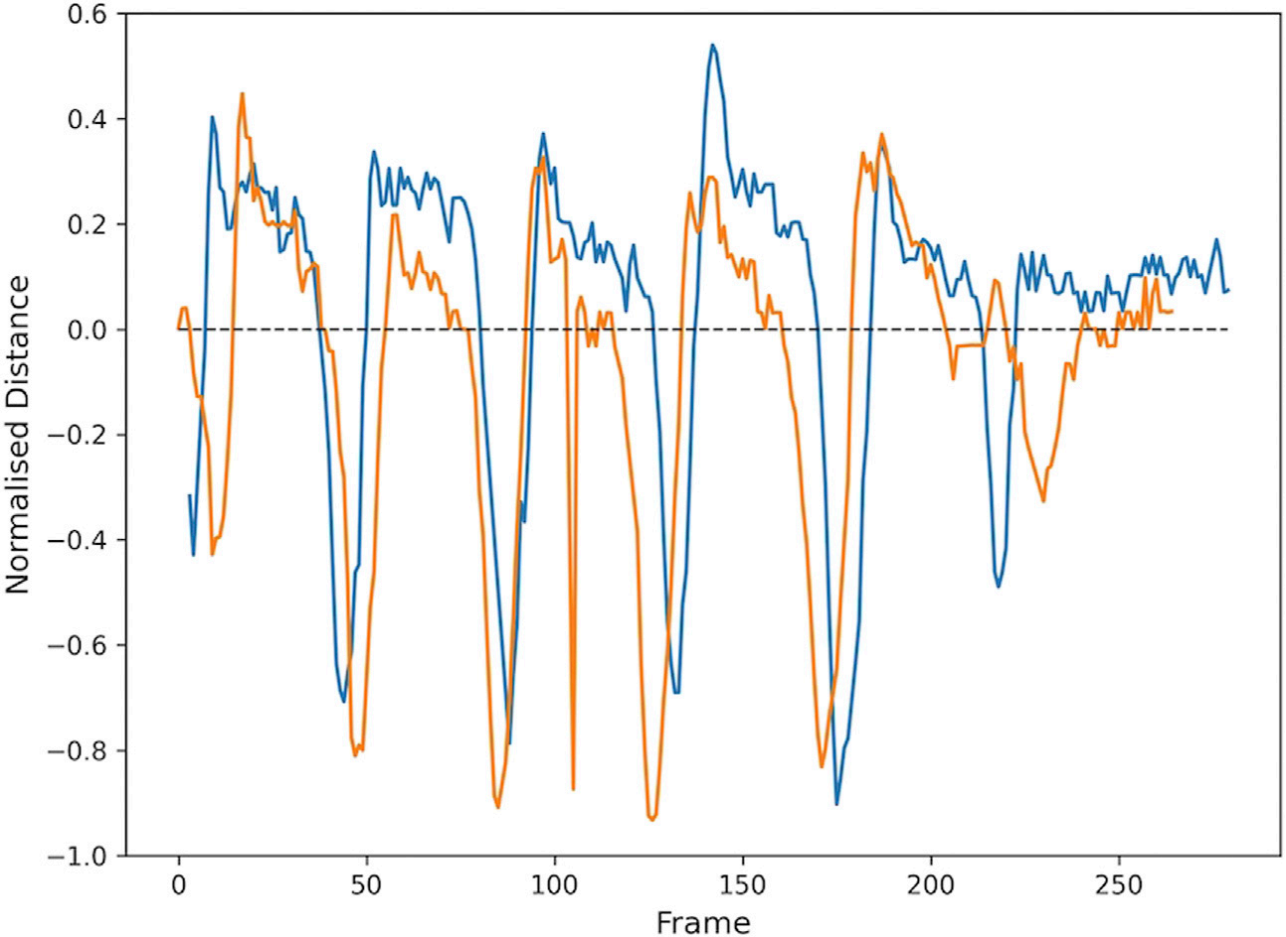
Vertical distance between left heel and toe over the duration of two separate videos from participant 1 (age 7). Oscillation peaks represent the alternate rising of the heel (below the dashed line) and toe (above the line). Distance decreases to zero as the foot becomes flat. Distance is normalised to the foot length.

Step length was calculated by measuring the horizontal distance from the right heel to the left heel (heel-to-heel distance) (Figure 2). This parameter was measured in videos taken from the side view and in which the camera moved alongside the participant. Due to the changing angle, it is not possible to measure this distance in videos in which the camera remains stationary and pans to keep the participant in view. In Figure 3, the distance is positive when the right heel is in front of the other, and negative when the right heel is behind. When the value is zero, the feet are beside each other. The peaks represent the length of each step. Lower leg length (from knee to ankle) was used to normalise the data. The results from two different videos from participant 1 are shown and illustrate good repeatability.

From the videos submitted, it is not possible to measure stride length, which would require the camera to remain stationary for the duration of the task. The set-up required to film the distance travelled in 10 steps, within the same view, limits practicality but it could be done.

#### 3.3.3 Foot drop (heel-toe distance)

Vertical heel-toe distance was also measured from the two side view videos in which the subject remains at the same angle to the camera. Figure 3 shows the vertical distance between the left heel and toes, normalised to foot length. Oscillation peaks represent the alternate rising of the heel (below the dashed line) and toe (above the line). Distance decreases to zero as the foot becomes flat. A value of minus one implies the heel is directly above the toe. A value of one implies the toe is directly above the heel. The results from two different videos from participant 1 are shown.

The step length and heel-toe alignment remain consistent across both videos from the same participant and could therefore potentially be used as parameters to measure progression of performance over time. Additional videos, and from a greater number of participants, are necessary to provide data on expected variability.

### 3.4 Hands-to-head while sitting

We received 10 videos from the front view and 10 videos from the side view that were suitable for analysis. The videos were from eight different participants. We received the most videos for this task, as this is the only task completed by all cohorts. The control participant uploaded an additional front and side view video of the task. All parameters described correspond to front-facing videos.

#### 3.4.1 Raised arm area

**FIGURE 4:**
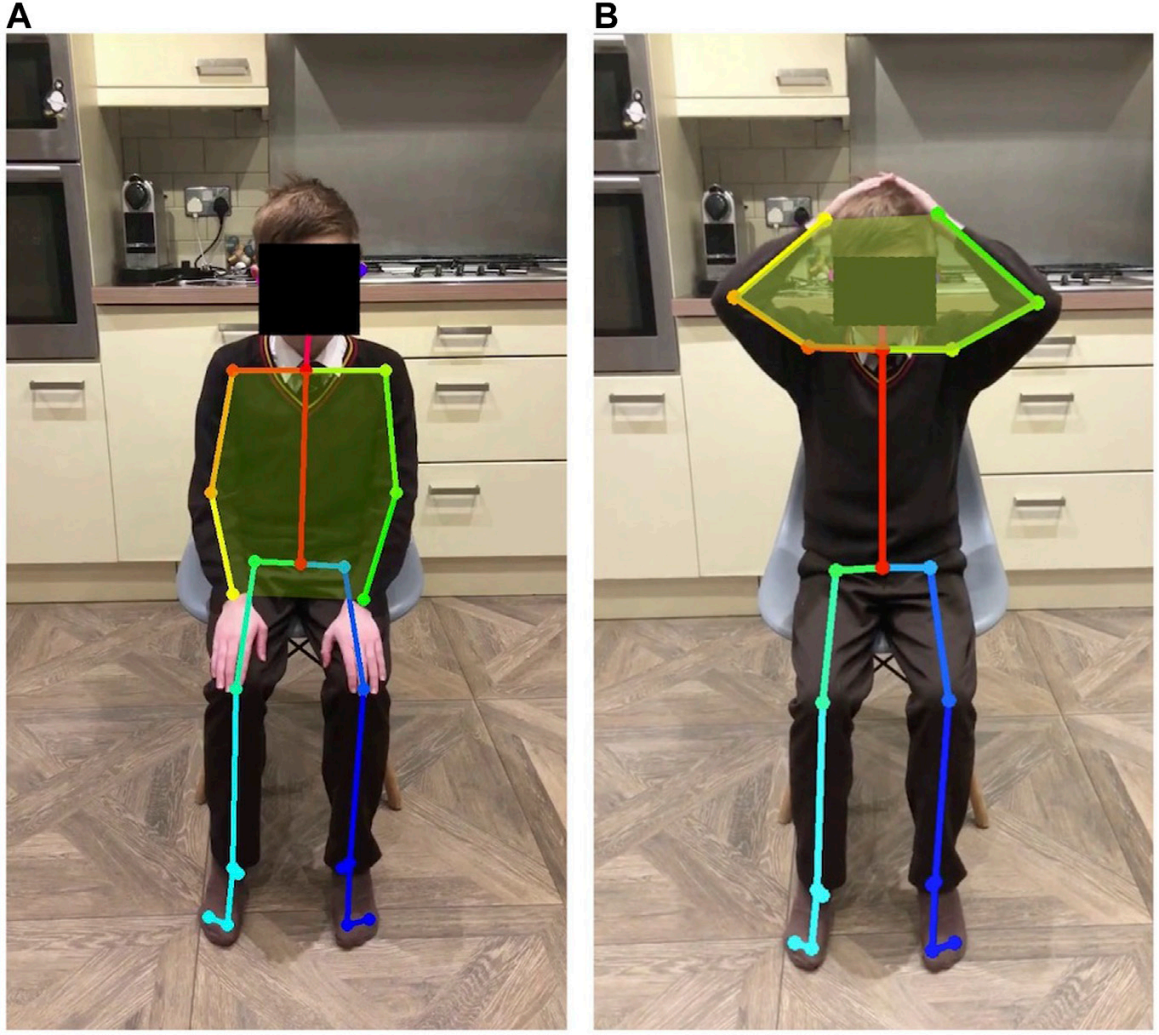
Body points captured by OpenPose show the area defined by shoulders, elbows and wrists when patient hands are in the resting position **(A)** and when hands are on the patient’s head **(B)**.

This task allows us to observe the placement of the elbows while the participant is touching their head; elbows pointing forward is an indication of arm weakness. The area between the two arms (the six-sided shape defined by the shoulders, elbows and wrists) is recorded when the hands are on the head and when the arms are at rest (Figure 4). The values are normalised by the area of the arms in the participant’s resting position. The normalised area when the participant’s hands are on their head is close to one (i.e., similar to the resting position) when their elbows are held open, and less than one if the elbows are pointing forward.

#### 3.4.2 Raised arm symmetry

**FIGURE 5:**
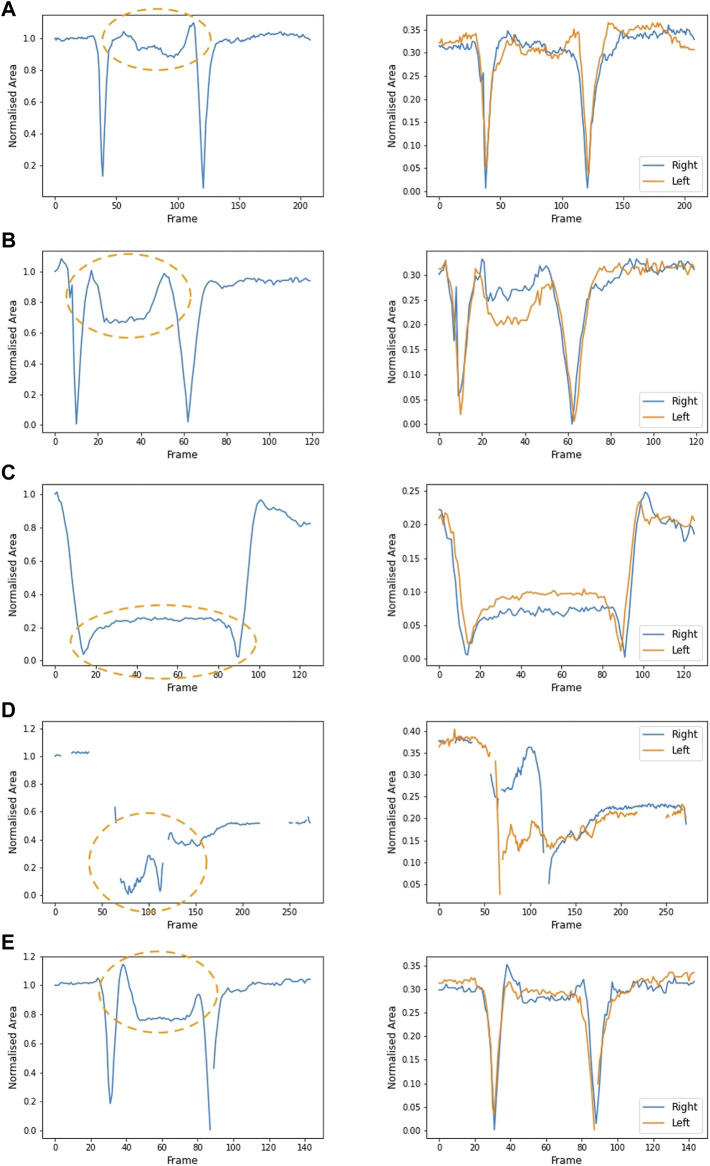
Area formed by the shoulders, elbows and wrists (left plot) over the duration of a video from five participants: **(A)** participant 1 (age 7), **(B)** participant 2 (age 13), **(C)** participant 8 (age 10), **(D)** participant 10 (age 15) and **(E)** control participant (age 9). Orange ovals show the part of the video where the hands are on the head. A larger value (between 0.8 and 1) illustrates that the elbows are open and wide. When the elbows are pointed forwards, the normalised area is smaller. Plotting the area formed by the right arm and left arm (the four-sided shape joining the shoulder, elbow, wrist and neck) illustrates the symmetry of movement (right plot). In both plots, the area is normalised to the area between the arms in the initial resting position. Participant 10 **(D)** lifted the right arm first, followed by the left arm. This lack of symmetry is evident in the right-hand plot.

**FIGURE 6:**
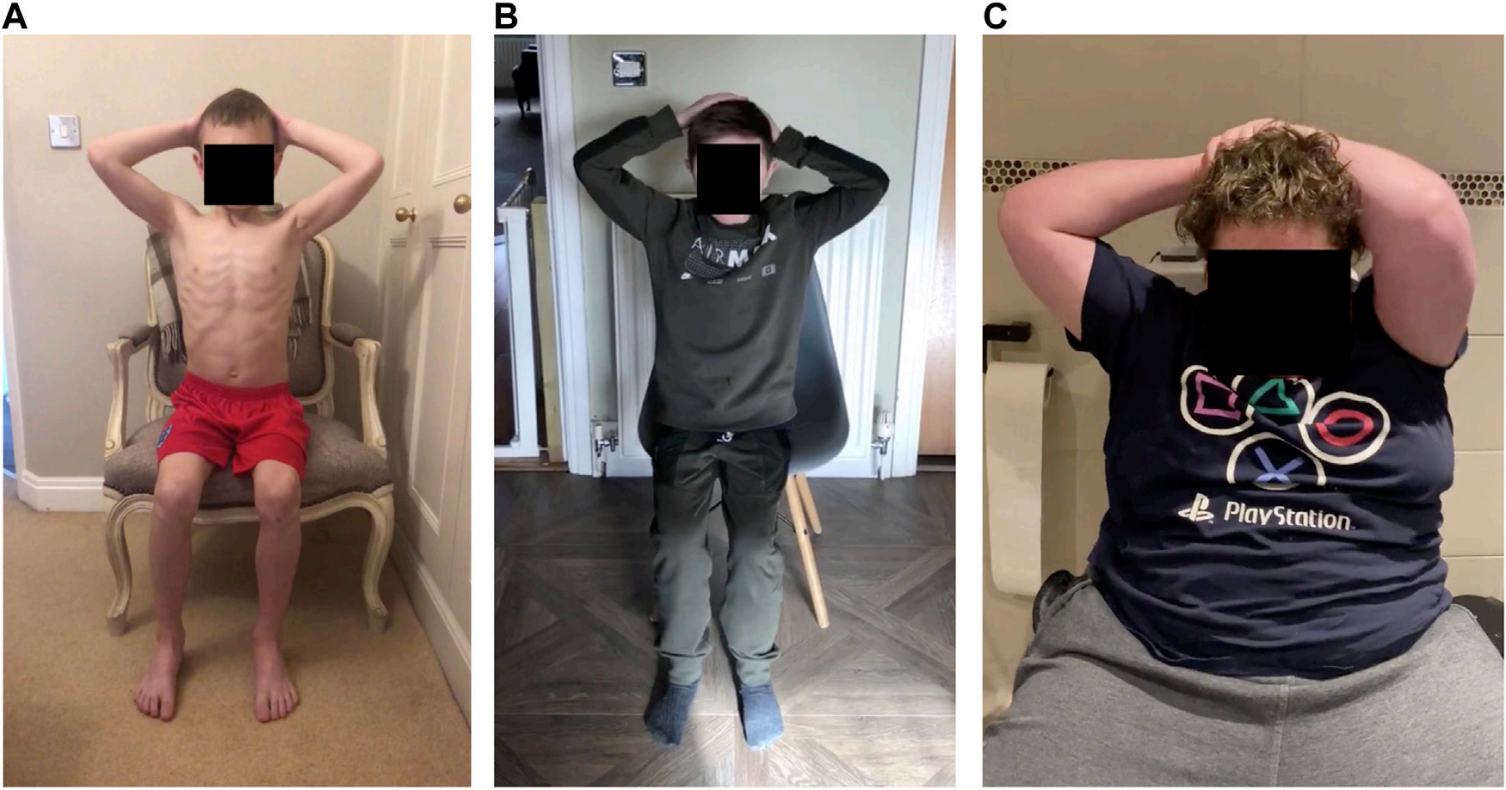
A frame from the hands-to-head while seated task for three participants: **(A)** Control participant (age 9), arms are relatively far back and symmetrical (Figure 5E). **(B)** Participant 2 (age 13), arms are reasonably far back, but the left hand is further down the head (Figure 5B). **(C)** Participant 10 (age 15) left arm is pointed forwards (Figure 5D).

Figures A–E also shows the area formed by each arm independently (right arm in blue and left in orange). Symmetrical movement is seen in A and E (control), with some small lack of symmetry in B and C. The participant in D lifted one arm at a time. Figures 5A–C show data from participants in cohort 1, aged 7, 8, and 10, respectively. Figure 5D shows data from a participant in cohort 3, aged 15. Of the participants in cohort 1, the arm movement is most symmetrical from the participant with the greatest raised arm area (Figure 5A). Symmetrical movement may be lost as disease progresses and muscle weakness is more evident (Figure 6).

#### 3.4.3 Lift motion: Time, smoothness and trunk movement

The relative time taken to lift and lower the arms can be analysed as a measure of strength, or control (Table 3). In participants where good symmetry was observed in the lift motion, the time taken to lift the arms is similar to the time taken to lower. The participant, who showed lack of symmetry for the arm lift, took a much shorter amount of time to lower the arms than to lift them, indicating a lack of control of the arms, which are dropped back down to the lap.

**TABLE 3:** Hands-to-head (while sitting): time taken to lift and lower arms.

Participant	Time to lift arms (s)	Time to lower arms (s)
1	0.8	0.7
2	0.7	1.0
8	0.9	0.8
10	0.8 (left), 1.2 (right)	0.3
Control	0.8	0.7

**FIGURE 7:**
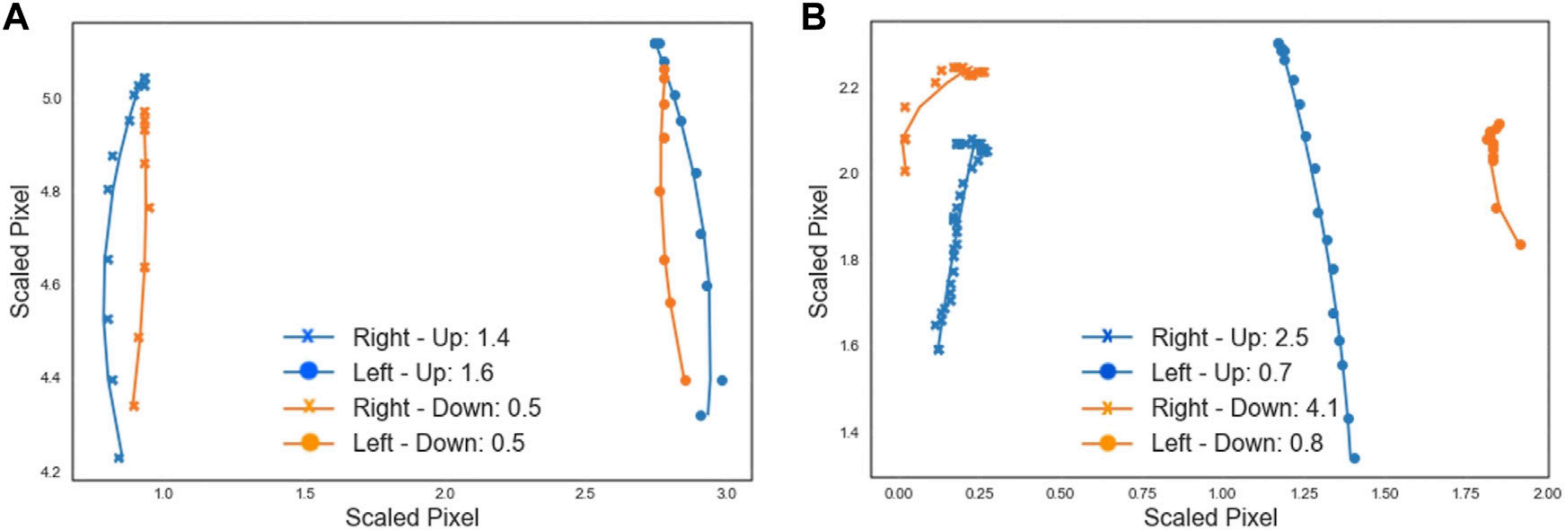
Each data point shows the scaled location of the left (circle) or right (cross) elbow in a frame of the video showing the lift (blue) or lower (orange) motion. A curve is fit to follow the trajectory and the plot legend shows the RMSE goodness of fit value associated with each curve, providing a measure of smoothness. **(A)** The video from participant 1 (age 7) shows reasonably smooth movement, with smoother movement on lowering than lifting. **(B)** The movement in the video from participant 10 (age 15) is relatively less smooth, particularly movement of the right arm. Pixel locations are scaled to allow for comparison across different videos.

Smoothness of movement is calculated by recording the trajectory of the elbows when the participant is lifting and lowering the arms. A curve is fitted to the trajectory (Figure 7), and the root-mean-square-error (RMSE) is calculated as a measure of how well the curve fits the data. The RMSE value for each curve shows the smoothness of the motion, where the lower the RMSE is, the smoother the arm movement was. Figure 7A shows lower RMSE values, meaning that the participant moved their arms in a smoother way, than the participant whose movement is shown in Figure 7B.

**FIGURE 8:**
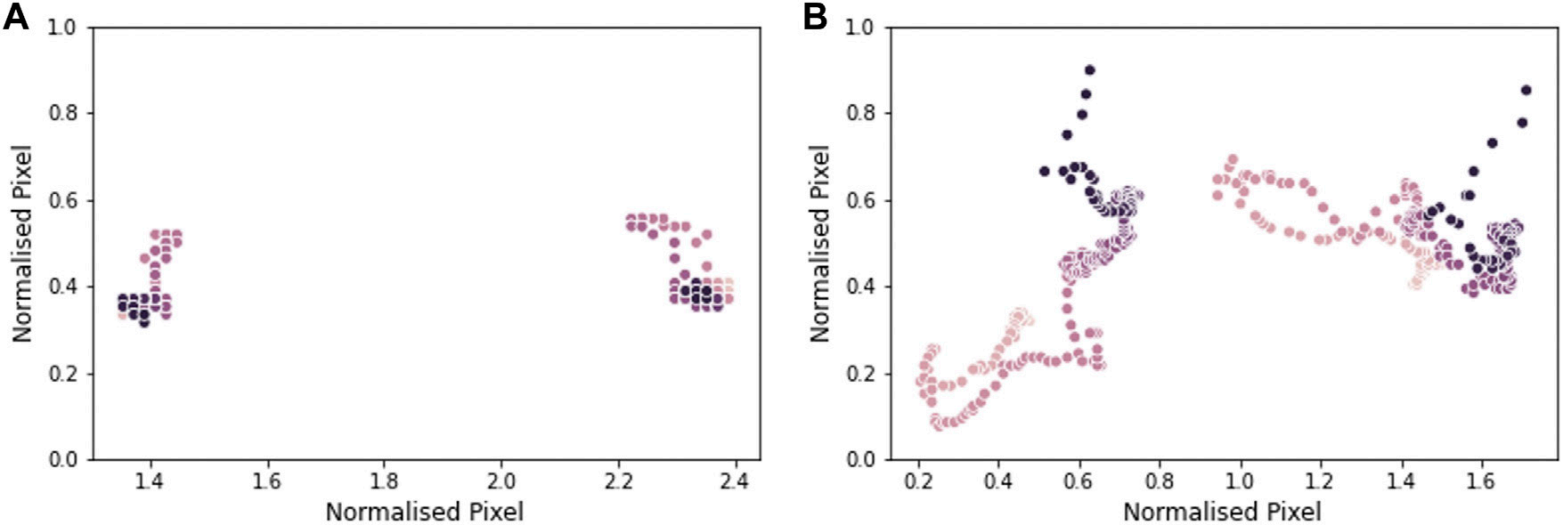
Shoulder movement in a video from participant 1 (age 7) in plot **(A)**, and a video from participant 10 (age 15) in plot **(B)**. Each data point shows the position of the shoulders in a frame of the video. The lighter the dot, the earlier in the video the data point is from. Pixel location is normalised by the distance between the shoulders.

Trunk movement was visualised by plotting the location of the shoulders over the course of the task. In Figure 8A, the shoulders remain relatively stable throughout the duration of the video, while Figure 8B shows a large amount of shoulder movement, as the participant used a rocking motion of the body to compensate for a lack of arm strength. Compensating for muscle weakness by changing the way a movement is performed is a common strategy in DMD as the disease progresses and muscle strength is progressively lost (Contesse et al., 2021).

### 3.5 Sit-to-stand followed by hands-to-head

We received five videos from the front view and four videos from the side view, from four different participants. We also received a front and side view video from the control participant. Comparison of the task performance between videos of different participants was difficult due to lack of regularity in the height and type of chair used, and the angle at which the video was taken. Again, these variations highlight areas that require further standardisation.

#### 3.5.1 Time

Not all participants completed both the front and side view videos, and participant 1 submitted two videos for each view. The difference in chair heights and styles makes direct comparison of times difficult.

We did not look at the total time taken to complete the task as here again there was lack of consistency in how the task was performed. Participants kept their hands on their heads for varying amounts of time. As no instruction was provided on this aspect, we cannot assess whether any pauses in the tasks are voluntary or not, and it is difficult to draw any conclusions from the total time taken.

#### 3.5.2 Leg alignment

**FIGURE 9:**
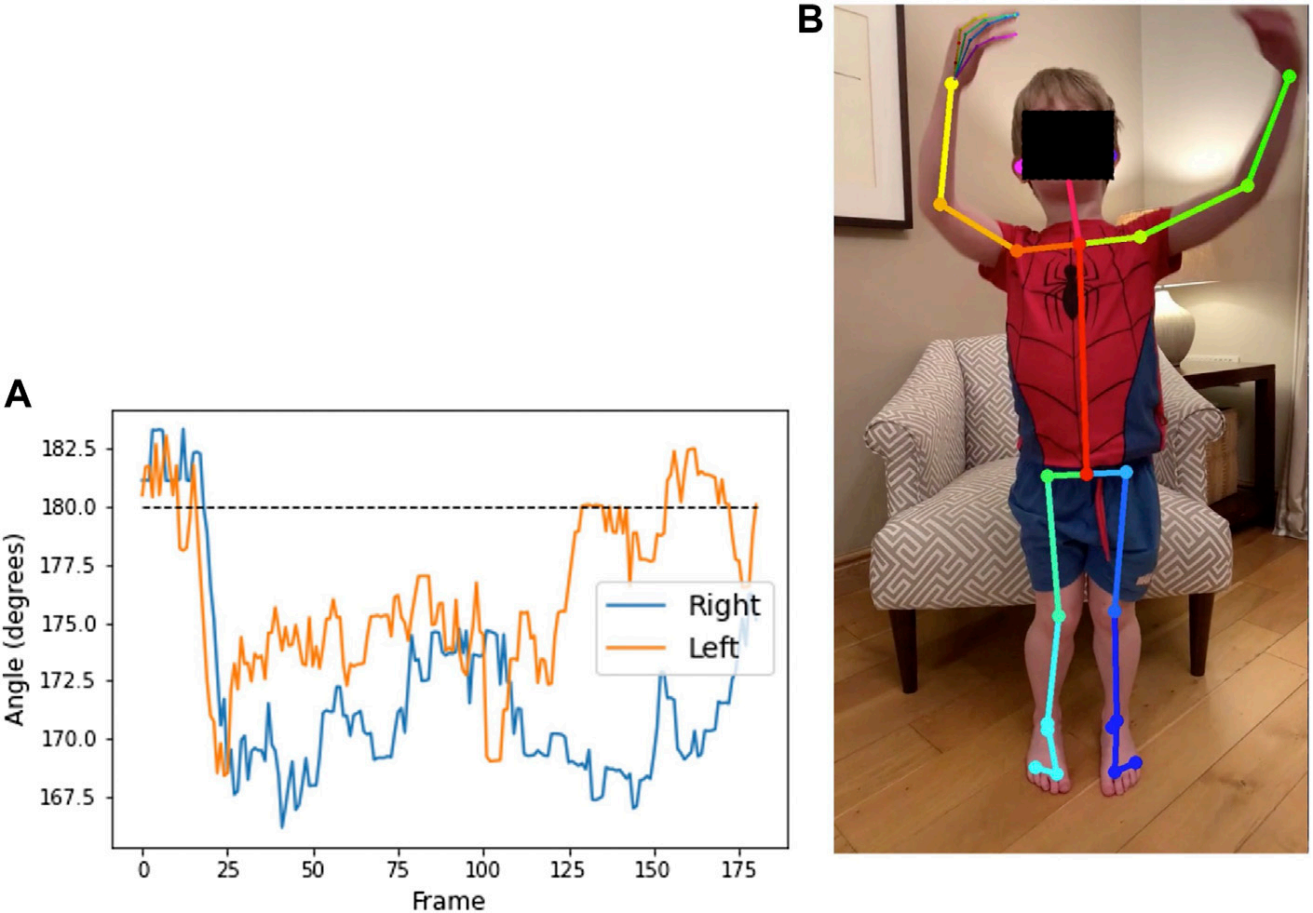
Plot **(A)** shows the angle at the outer knee over the duration of a video from participant 1 (age 7) completing the sit-to-stand test. Angle of the legs relative to the camera makes the value at the start and end of the video, while seated, difficult to interpret. The region from frame 30 to frame 120 shows the duration of the standing phase. The dashed line illustrates that the legs are straight. Below the dashed line implies that the knees are Q20 pointed inwards, as seen in the annotated image of frame 44 **(B)**.

Figure 9 shows the alignment of the hip, knee, and ankle when viewed from the front, for a video from participant 1. The point of interest is whether the knees move together when standing up, as a means of compensation. A value of 180° on the plot implies that the legs remain straight when standing. When the value is below 180, the knees are angled inwards. For this participant, we see that the knees move together slightly as he stands.

#### 3.5.3 Foot position

Foot-to-foot distance and foot alignment were measured, from front-view videos, to identify any alterations in stance when standing up from seated. The horizontal distance in pixels is measured between heels and between toes, then normalised by knee to ankle distance. The heel-toe alignment is the horizontal distance between the heel and the toe on each foot. A value of zero implies the heel is directly behind the toe, the alignment is negative when the toes are turned outwards, and positive when the toes are turned inwards.

**FIGURE 10:**
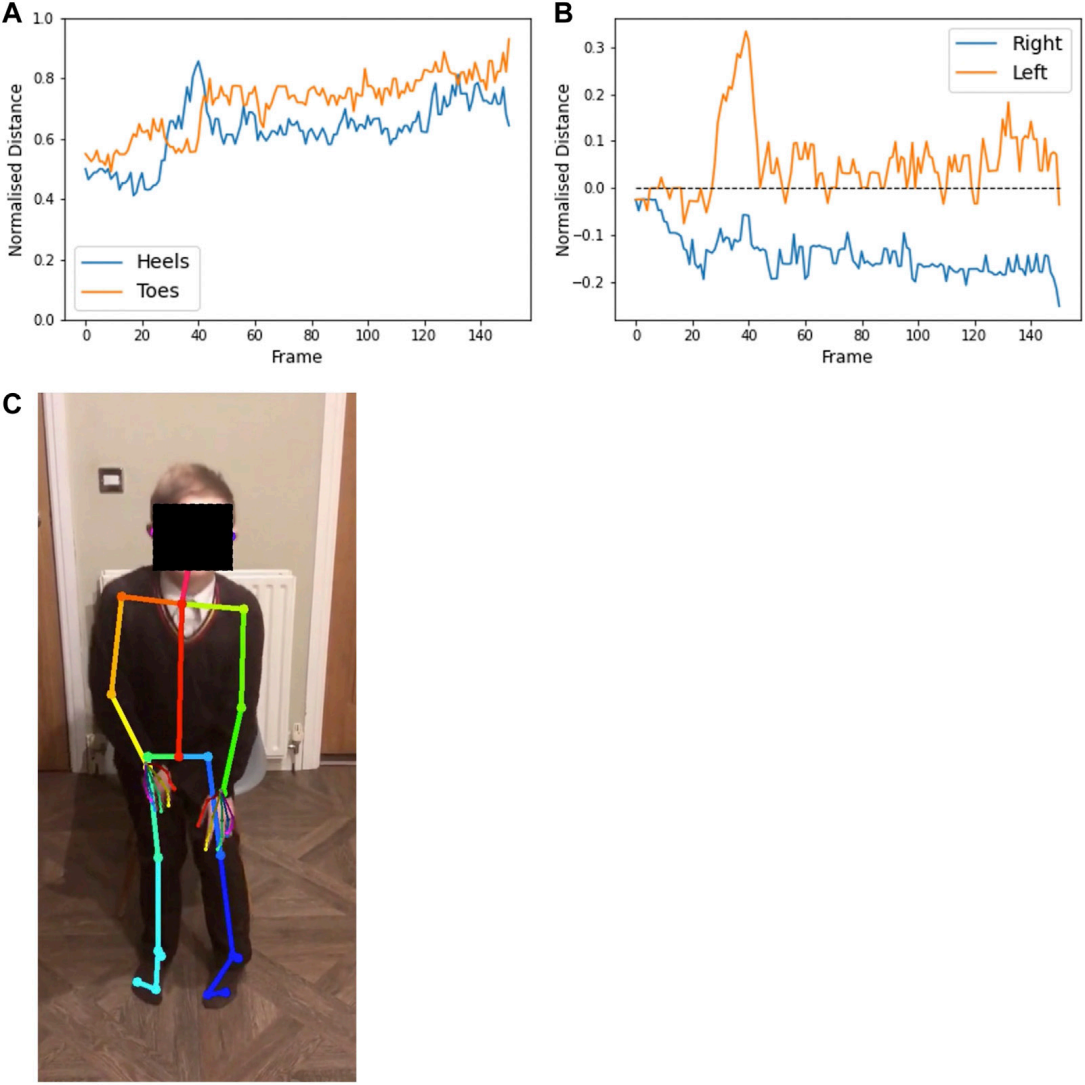
Plot **(A)** shows the normalised distance between the heels (blue) and big toes (orange) throughout a video from participant 2 (age 13) completing the sit-to-stand test. Plot **(B)** shows the horizontal distance between the heel and toe of each foot (values are positive when the heels are pointing outward relative to the toes), with the right foot (blue) remaining toes turned out (below the dashed line) and the left foot (orange) tending to have toes turned in. The feet remain relatively close together, but the left heel slides outwards [as seen in the annotated image of frame 38 **(C)**] when the participant stands up. Distances are normalised by the lower leg length.

Figure 10 shows the plots of these measures for a video from participant 2. In the heel-toe alignment plot, the compensatory movement of the left heel as he stands up is particularly clear.

### 3.6 Usability of the app

Participants were asked to complete a feedback survey that contained 21 questions around three main topics; whether the app was user-friendly and accessible, the quality of the technical support received and the clarity of the DMD-QoL. All respondents agreed that the app was easy to download and easy to navigate. 66.7% said the app was “user-friendly,” whilst the remaining 33.3% said it was “somewhat user friendly.” 50% of respondents used the app daily, whilst the other 50% said they used the app a few times over the 7-day testing period, citing issues with updating and task notifications disappearing. One user (16.6%) said they did not use the app daily due to getting reminders at an inconvenient time, suggesting that improvements to the timing or configurability of notifications can be made. Regarding these and other technical issues, all participants said questions and concerns about the app were addressed or resolved by the technical support timely and effectively. Participants also mentioned that a clearer guide on how to film the videos would help.

On the app content and instructions, 83.4% said that the video tasks and the instructions for the tasks were easy to understand, with the remaining 16.6% saying that they were somewhat easy to understand. 83.4% said their performance in the tasks were representative of their physical abilities whilst the remaining 16.6% said they were somewhat representative. Whilst 83.4% is very high there is an overlap of parameters captured by the tasks and therefore this will need to be refined in future validation studies.

### 3.7 Preferred format of the DMD-QoL questionnaire

Five participants were given the “Scroll” format and five received the “Next” format. All 10 participants completed and submitted the questionnaire.

The participants using the “Scroll” format took on average 1 minute and 59 s. This average was found by removing three outliers: 26 min, 17 h (16:41:04) and 10 h (10:14:17). These longer times were removed because, likely, the questionnaire had not been completed in one sitting. The “Next” format had an average time of 4 minutes and 27 s, with no obvious outliers, showing that the “Next” format took longer to complete. This was confirmed during a debrief meeting in which it was mentioned that “scrolling was quicker and easier.”

## 4 Discussion

This project showed the feasibility of home video capture and computer vision analysis, using an integrated data platform, for the assessment of outcomes related to lower and upper limb function in DMD patients.

We showed the usability of the DMD Home app as a method to video record motor tasks, deliver instructions to participants, and to collect patient-reported outcomes (e.g., DMD-QoL). However, we acknowledge several limitations which are later described. We also demonstrated that computer vision analysis with OpenPose allowed us to characterise movement in an objective manner and using parameters other than conventional clinical assessments. While the sample size was small and participants were self-assigned to the different cohorts, hence not having a representative sample of all DMD stages, we could still extract highly relevant information particularly from upper body tasks from participants who were in the ambulant and non-ambulant stages of the disease.

### 4.1 Upper limb function

The hands-to-head functional task was performed by both ambulant and non-ambulant participants, and while not equivalent, it may align with PUL items in the shoulder high level and elbow middle level. The number of videos available for analysis was larger in cohort 1 than in cohort 3. This task allowed us to quantify the area defined by the shoulders, elbows and wrists, at rest and when the participant’s hands were on their head. Normalised areas smaller than one show that elbows are pointing forward, an adjustment made to compensate for arm weakness. Figure 5A shows the plot from a 7-year-old participant with an area value close to one, while in Figure 5C the area value of a 10-year old participant is 0.2, and in Figure 5D the area could not be calculated as the participant was unable to perform the task (15 years of age). Although the lack of standardisation in the video capture angle makes accurate comparison across videos difficult, the reduction in the measure between Figures 5A–D correlates with a clear observed difference in the performance. Figure 6 shows pictures of the control participant and participants 2 and 10. This type of analysis could be used to detect disease progression through small variations of the area value. Even if exploratory, these data seem to show a progressive reduced ability to perform the task as participants grow older.

Computer vision techniques also allowed analysis of dynamic parameters like symmetry of movement. Graphs of the same participants (Figure 5) show how symmetry of movement may be lost with disease progression and increased muscle weakness.

Time taken to lift and lower the arms could be analysed as a measure of strength or control and the measure correlated well with symmetry, with similar time taken for both arm lift and lowering in participants with good symmetry of movement. The participant who showed lack of symmetry showed shorter time to lower the arms (reduced control) and longer time to lift the arms (reduced strength).

Dynamic measures such as smoothness (Figure 7) could be useful measures of arm movement control or strength, with lower RMSE values indicating smoother arm movement. In a participant with asymmetrical arm movement, RMSE values were different for each arm. Measuring both symmetry and smoothness of movement would better characterise strength and control. Correlation with measures of muscle strength would be needed in future validation studies.

Understanding, characterising and accurately measuring compensatory movements to make up for the loss of motor function in DMD patients is important, as they may indicate disease progression and trigger the start of use of assistive devices and other aids (Sá et al., 2016). Additionally, increased trunk movement can be observed over a 6-month period despite the fact essential motor tasks are preserved (Maciel et al., 2021a; Maciel et al., 2021b), indicating the importance of the early detection and quantification of changes in compensatory movements. We assessed trunk movement by measuring the location of the shoulders over the course of the hands-to-head while seated task, which remained quite stationary in participant 1 (ambulant) compared to participant 10 (non-ambulant). While there are scales accounting for the number and type of compensatory movements that patients do when performing a functional task (Contesse et al., 2021), there are no current methods to quantify them easily. Currently quantification of trunk movement has been possible with the use of clinical evaluation tools requiring the intervention of external evaluators (Sá et al., 2016) or through complex optical motion capture technology (Peeters et al., 2019).

Quantification of upper limb function has been tested by measuring the reachable workspace using a single stereo camera that tracks the location of different body landmarks with small LED markers applied to the skin or clothes (Kurillo et al., 2012). While promising, this approach showed limitations in detection and tracking of the markers, inability to discriminate between compensatory movements and use restricted to clinical settings. Video capture through a smartphone app followed by computer vision analysis is user-friendly and allows monitoring and quantification of small changes in movement trajectories, as well as upper body compensatory strategies.

### 4.2 Lower limb function

Timed sit-to-stand tests have been used to assess functional muscle strength of the lower limbs in children with cerebral palsy (Wang et al., 2012; Kumban et al., 2013) and to assess motor performance in children with cri du chat syndrome, a rare genetic condition that shows weakened muscle tone (Abbruzzese et al., 2020). Although it has not been extensively explored in DMD (Hukuda et al., 2017), its potential utility to assess the transfer stage has been suggested, as sitting down and rising from the floor seems to be lost before the loss of ambulation in DMD patients (Sá et al., 2016).

From the front view of the sit-to-stand videos, leg alignment, foot-to-foot distance and foot alignment were measured over the course of the task. Variations in these parameters provided information about compensatory movements made by DMD patients when standing up from a seated position. Inward movement of the knees and toes when standing up could be a mechanism to compensate for reduced lower limb strength. Further characterization of compensatory movements while standing from a seated position requires larger participant sample and longitudinal data collection.

### 4.3 Limitations

This project to co-design a suitable solution had several limitations. Participants were restricted to United Kingdom English speaking only. Lack of standardisation in different aspects of the performance of the tasks and recording of the video was a main limitation that needs to be addressed for future studies.

Only minimal instructions to conduct the tasks were provided, which resulted in different techniques for task performance among the participants, meaning comparisons across participants doing the same task could not always be made. Although such comparison was not the objective of this project, precise instructions would be needed if these assessments were to be further validated for inclusion in future clinical trials.

The limited guidance provided also resulted in inconsistent filming techniques and the filming angle not being adapted to the task or the parameters measured, which made it difficult to analyse the same parameters across the different videos (e.g., step length). For example, stride length (common gait endpoint) could not be measured as the camera was moving alongside the participant, while it would need to have remained stationary and placed further away from the boy to measure the full stride.

The height of the chairs used in the sit-to-stand task was variable among participants. Lack of standardisation of chair height limited the range of parameters that could be consistently measured (e.g., time taken to stand up). A height-adjusting stool might be advised to allow a 90-degree hip-knee angle, as a greater angle facilitates standing up more quickly.

Another aspect that we did not pursue was the performance of tasks aligned with essential activities of daily living, such as eating, drinking or reaching for an object, which are covered in other types of scales and assessments (Mayhew et al., 2013; Landfeldt et al., 2015; Contesse et al., 2021). However, it can be presumed that pose estimation analysis could provide further insights of the quality of movement in those situations and allow for detection of new parameters to assess mobility in real-life conditions.

We wanted to explore tasks that could best describe the transition from the ambulant to the non-ambulant stage, hence the choice of the “sit-to-stand followed by hands-to-head while standing” task. However, there were no participants assigned to cohort 2 (“requires support to walk but can stand independently”). As only ambulant participants were able to carry out the task, we did not gain more insight from their performance of the “hand-to-head while standing” portion, compared to the “hands to head while seated” task. During the transfer stage, the ability to stand from supine and to walk/run 10 m are lost, but the patient is still able to remain standing (Broomfield et al., 2020). The tasks “sit-to-stand” and “hands-to-head” (sitting/standing) either evaluated together or as separate tests, merit further investigation to identify digital endpoints to assess the transfer stage. The combination of static (e.g., leg alignment) and dynamic (e.g., smoothness) parameters that can be analysed through computer vision techniques allows more granular insights into movement control and strategies to compensate for reduced function and muscle weakness.

We were not able to accurately ascertain the stage of the disease, as no validated instrument was administered to assign the participants into the different cohorts. In addition, the age of the participant at diagnosis was not collected, nor if they were treated with corticosteroids or other medicines.

### 4.4 Validation

The project showed the feasibility of digital remote assessment of functional tasks, and the ability of computer vision analysis for capturing data that could be used towards clinical trial endpoints to convey how the patient functions or feels in the home environment. Both analytical and clinical validation of this approach are required and include further refinement of the tasks and standardisation of their performance to ensure consistent and reproducible measurements; correlation of the video analysis endpoints with validated functional and muscle strength measurements and data collection from normative controls from the same age range as DMD participants. In this approach, measures can be developed to score, objectively, the tasks according to key movement parameters (e.g., raised arm area, symmetry and smoothness of movement) and compensatory strategies (e.g., leg alignment, trunk movement), to provide an automated, quantitative assessment of patient motor function. This automated scoring system would allow a more comprehensive, unbiased, characterisation of both upper and lower limb function in patients who are transitioning to non-ambulation.

While the use of key standardised tasks is essential to demonstrate the validity and reliability of this new approach, another aspect we would like to evaluate is this application of this approach to the analysis of natural movements to measure disease progression. Further validation efforts should be targeted to capture and analyse participant movement performed in a real-world setting and while carrying out daily life activities. Measuring disease progression, stabilisation or improvement after therapeutic intervention using home digital assessments and AI-based analysis would ensure outcome measures that are child-friendly, easy to do and that minimally interfere with their daily routine. Such data would fill the current lack of robust, validated real-world evidence required for HTA and reimbursement decisions.

## 5 Conclusion

We have shown the usability and potential clinical utility of the DMD Home-based video capture and computer vision analytics prototype for the assessment of motor function in DMD patients. Video technology offers the possibility to perform clinical assessments and capture how patients function at home, causing minimal disruption to their lives. Video capture linked with computer vision analytics may allow the identification of sensitive digital endpoints that could potentially be used in clinical research. Validation of this approach is critical to identify new meaningful outcome measures that remove the subjectivity of traditional assessments and that can be used in the evaluation of future innovative therapies.

## Data Availability

The raw data supporting the conclusion of this article will be made available by the authors, without undue reservation.

## Footnotes

1 Aparito Patient Group Accelerator Programme. Available at: https://www.aparito.com/patient-group-programme/[Accessed 11 March 2022].

2 Aparito Digital Endpoints Patient Group Accelerator Programme—first two organisations selected. Aparito Blog. Available at: https://www.aparito.com/aparito-digital-endpoints-patient-group-accelerator-programme-first-two-organisations-selected/[Accessed 11 March 2022].
